# Genome-Based Analysis of Extended-Spectrum β-Lactamase-Producing *Escherichia coli* in the Aquatic Environment and Nile Perch (*Lates niloticus*) of Lake Victoria, Tanzania

**DOI:** 10.3389/fmicb.2020.00108

**Published:** 2020-02-21

**Authors:** Zebedayo Baniga, Yaovi M. Gildas Hounmanou, Egle Kudirkiene, Lughano J. M. Kusiluka, Robinson H. Mdegela, Anders Dalsgaard

**Affiliations:** ^1^Department of Veterinary Medicine and Public Health, College of Veterinary Medicine and Biomedical Sciences, Sokoine University of Agriculture, Morogoro, Tanzania; ^2^Department of Fisheries Development, National Fish Quality Control Laboratory-Nyegezi, Mwanza, Tanzania; ^3^Department of Veterinary and Animal Sciences, Faculty of Health and Medical Sciences, University of Copenhagen, Copenhagen, Denmark; ^4^Mzumbe University, Mzumbe, Tanzania; ^5^School of Chemical and Biomedical Engineering, Nanyang Technological University, Singapore, Singapore

**Keywords:** nile perch, food safety, extended-spectrum β-lactamase, antimicrobial resistance, *Escherichia coli*

## Abstract

Extended-spectrum β-lactamase (ESBL)-producing bacteria constitute an emerging global health issue with food products being vehicles of transmission and the aquatic environments serving as potential reservoirs. This study aimed to characterize ESBL-producing *Escherichia coli* in Nile perch and water from Lake Victoria in Tanzania. A total of 180 samples of Nile perch and 60 water samples were screened for ESBL-producing *E. coli* on MacConkey agar supplemented with 2 μg/ml of cefotaxime and confirmed by *bla*_CTX–M_ and *bla*_TEM_ PCR. Antimicrobial resistance was determined by the disk diffusion method, and the ESBL-producing isolates were whole genome sequencing (WGS). ESBL-producing *E. coli* were detected in eight of the 180 analyzed Nile perch samples, and only one water sample was positive (1.7%, *n* = 60). Isolates were resistant to sulfamethoxazole–trimethoprim (100%), ampicillin/cloxacillin (100%), erythromycin 72.7% (8/11), tetracycline 90.9% (10/11), and nalidixic acid 63.6% (7/11). This mostly corroborates the resistance genes that they carried for sulfonamides (*sul*1 and *sul*2), trimethoprim (*dfr*A and *dfr*B), aminoglycosides [*aac(3)-IId*, *str*A, and *str*B], tetracycline [*tet*(B) and *tet*(D)], and fluoroquinolones (*qep*A4). They harbored plasmid replicon types IncF, IncX, IncQ, and Col and carried *bla*_CTX–M–__15_ and *bla*_TEM–__1__B_ genes generally found on the same contigs as the IncF plasmid replicon. Although epidemiologically unrelated, the strains formed three separate sequence type–phylogroup–serotype-specific clusters: C1, C2, and C3. Cluster C1 included five strains (3 to 13 SNPs) belonging to ST167, phylogroup A, and serotype O9:H21; the two C2 strains (11 SNPs) belong to ST156, phylogroup B1, and serotype ONT:H28; and C3 was made up of four strains (SNPs ranged from 4 to 17) of ST636, phylogroup B2, and serotype O45:H7. The common virulence gene *gad* was reported in all strains. In addition, strains in C2 and C3 possessed *iss*, *lpf*A, and *nfa*E virulence genes, and the *vat* gene was found only in C3. The present study reports the occurrence of multidrug-resistant ESBL-producing *E. coli* carrying plasmid-mediated ESBL genes in offshore water and Nile perch in Lake Victoria. Strains formed three clonal clusters of unknown origin. This study reveals that the Lake may serve as reservoir for ESBL-producing bacteria that can be transmitted by fish as a food chain hazard of One-Health concern.

## Introduction

*Escherichia coli* and other related bacteria can produce extended-spectrum β-lactamase (ESBL) enzymes that hydrolyze a broad spectrum of β-lactam drugs such as cephalosporins (e.g., ceftazidime and cefotaxime) and monobactams (e.g., aztreonam and nocardicin), classes of antimicrobials that are critical in human medicine (Lavilla et al., 2008; Shaikh et al., 2015; Adelowo et al., 2018). ESBL-producing Gram-negative bacteria especially those producing cefotaximase-Munich (CTX-M) enzymes have emerged as important pathogens causing healthcare- and community-associated infections worldwide.

Studies in many different countries increasingly document how foods may be important sources of ESBL-producing *E. coli*, such as vegetables, poultry, pork, and other animal foods. In particular, poultry meat has been reported commonly to be associated with ESBL-producing *E. coli* globally (Overdevest, 2011; Chishimba et al., 2016; Nguyen et al., 2016; Falgenhauer et al., 2019; Projahn et al., 2019). Possible horizontal transfer of resistance genes from urban sewage and clinical isolates to bacteria associated with fish and aquatic recipients of wastewater has been documented (Kümmerer, 2009; Martinez, 2009; Jiang et al., 2013; Blaak et al., 2014).

The β-lactamase enzymes are derived from mutations in temoneira (TEM), sulfhydryl variable (SHV), and CTX-M genes located on bacterial plasmids or chromosomes (Ben Said et al., 2015; Legese et al., 2017; Adelowo et al., 2018). These genes can easily be horizontally transferred from one bacterial strain to another including across bacterial species (Lavilla et al., 2008). The SHV enzyme seems more dominant in *Klebsiella pneumoniae* among the Enterobacteriaceae (Rupp and Fey, 2003). The CTX-M enzymes are often the most common ESBLs produced by *E. coli* and have been isolated in human clinical, animal food, and environmental samples (Egea et al., 2012; Boonyasiri et al., 2014; Dib et al., 2018). On the other hand, the TEM enzymes especially of TEM-1 and TEM-2 are predominantly reported in *E. coli* and other members of Enterobacteriaceae, although TEM-1 is not considered as ESBLs. These TEM enzymes have been reported from *Klebsiella* spp. and *Enterobacter* spp. isolated in food, clinical, and environmental samples around the world as previously described (Smet et al., 2010; Delgado et al., 2016).

The reservoirs of ESBL-producing *E. coli* are warm-blooded animals including humans, which can transmit the bacteria to different environments, for example, aquatic environments, through fecal pollution (Jiang et al., 2012). Recently, studies have reported ESBL-producing *E. coli* in seafood such as shrimps, sardines, farmed fish, Nile tilapia (*Oreochromis niloticus*) as well as in frozen mackerel (Jiang et al., 2012; Moremi et al., 2016b; Nasreldin and Khaldoon, 2015; Dib et al., 2018). It has been documented that urban sewage, for example, from hospitals, is an important source of ESBL-producing enteric bacteria (Ojer-Usoz et al., 2013; Abgottspon et al., 2014). Thus, ESBL-producing bacteria in sewage and in runoff water from agricultural soil fertilized with livestock manure can enter the aquatic environment where antimicrobial resistance genes may be transferred horizontally to the ubiquitous bacterial flora, although the rate and health importance of such transfer are unknown.

In Tanzania, ESBL-producing *E. coli* have been isolated from different sources such as human specimens, foods, and aquatic environments (Moyo et al., 2010; Moremi et al., 2016b). Although ESBL-producing *E. coli* have been reported in humans in different regions of Tanzania (Moyo et al., 2010; Seni et al., 2016; Katakweba et al., 2018) and also in livestock and poultry (Katakweba et al., 2018), the importance of livestock and their meats as sources of ESBL-producing *E. coli* is unknown. ESBL-producing Enterobacteriaceae recovered from the aquatic environments and tilapia from Lake Victoria were previously characterized (Moremi et al., 2016b). This study was carried out with tilapia, which is a fish species commonly found in shallow water, which often is polluted by discharged human wastes of different sources mostly of fecal origin. Thus, a variety of ESBL-producing bacteria were found in the samples analyzed by Moremi et al. (2016b). Our study investigated the occurrence of ESBL-producing Enterobacteriaceae spp. along the chain from capture to market including water and Nile perch at offshore deep water fishing areas. We further applied whole genome sequencing (WGS), which provides important information about antimicrobial resistance and virulence genes in bacterial pathogens and is a powerful molecular tool in investigations of disease outbreaks.

The aim of this study was to determine the occurrence and genomic characteristics of ESBL-producing *E. coli* isolated in water at offshore fishing grounds and in Nile perch (*Lates niloticus*) from Lake Victoria, Tanzania.

## Materials and Methods

### Study Design, Sample Collection, and Processing

The study employed a cross-sectional design and was conducted from February to July 2017. A total of 240 samples of water and Nile perch were collected and analyzed for ESBL-producing Enterobacteriaceae, and the isolates were tested for antimicrobial resistance. This sample size was calculated based on the estimated prevalence of ESBL-producing Enterobacteriaceae spp. detected in other fish spp. from Lake Victoria reported by Moremi et al. (2016b) using the following formula:

$$n={\left(Z_{\alpha }\right)}^{2}\times P\,\left(1-P\right)\,d^{2}$$

Out of 240 samples, 180 were Nile perch and were collected from fishing grounds (*n* = 60), landing sites (*n* = 60), and domestic fish markets (*n* = 60). In addition, offshore water samples (*n* = 60) were collected and analyzed from fishing grounds. The size of fish sampled ranged between 1 and 2 kg by weight. The selection of Nile perch samples at markets was based on the availability of fish from vendors. At each market, 10 samples were collected from five retailers, with two fish purchased from each vendor. We did not register how many Nile perch were on sale at each market. Details on sample collection, transport, and processing are provided by Baniga et al. (2019). Nile perch samples were prepared by weighing intestines, gills, flesh, and fish surface mucus in a ratio of 1:9 to Buffered Peptone Water (BPW) (Oxoid Ltd., Hampshire, United Kingdom). Fish intestines, gills, and flesh (15- to 25-g volumes) were homogenized for 60 s in a stomacher (Seward 400, United Kingdom), whereas fish mucus was obtained and analyzed by massaging the fish surface in a sterile stomacher bag containing 225 ml of BPW. The weight of fish gills and intestine samples was not always the same because the weight varies based on the size of the fish; for example, a fish weighing 1 kg will have gills and intestines each weighing below 25 g and not less than 15 g. Stomacher bags used to obtain the surface mucus of fish were of 16063/0606 size, that is, Stomacher^®^ Bags, Seward Genuine 16063/0606.^1^ The collection of fish mucus sample was done while fish was still intact before other subsamples, that is, flesh, gills, and intestines, were collected. All fish samples were processed following standard method (ISO 6887-3,2003). Twenty-five milliliters of each water sample was filtered using a standard filter membrane of 0.45 μl of pore size (Thermo Fisher Scientific, Waltham, MA, United States). After filtration, the filter membrane was torn into pieces using sterile forceps and then placed into 225 ml of BPW, which were shaken rigorously and then incubated at 37°C overnight for enrichment before plating out on the MacConkey agar. Thus, the limit of detection was 4 colony-forming units (cfu) ESBL-producing *Escherichia coli* per 100 ml. Water sample handling and processing were done according to ISO and Tanzanian standards incorporated with some modifications (ISO 9308-1,2000; Tzs 117,1981).

### Study Site

The study was conducted along the Tanzanian basin of Lake Victoria in Mwanza. Sampling points included fishing grounds (open water), landing sites, and domestic fish markets located in Ilemela and Nyamagana districts in the Mwanza region. At the fishing grounds, there were no other activities besides fishing, and there were no toilet facilities on board. At some of the landing sites, there were households nearby and a range of different human activities including agriculture and scavenging animals, for example, cattle, goats, and dogs, around the landing sites. Also, there were several migratory fish-eating birds around the shore of the lake. At every landing site, there were toilet facilities, but it is uncertain whether these discharged into the lake. In the domestic markets, a variety of goods were sold, and toilets were present at all markets visited during sampling. However, there was poor waste management around the markets owing to accumulation and access of wastes in the garbage bins and also lack of proper wastewater discharge. Processing of samples and bacteriological analysis were done at the National Fish Quality Control Laboratory, Mwanza, and at the laboratory of the Department of Microbiology, Parasitology and Biotechnology, Sokoine University of Agriculture (SUA). The WGS of ESBL-producing *E. coli* was done at the Department of Veterinary and Animal Sciences, University of Copenhagen, Denmark.

### Identification of Extended-Spectrum β-Lactamase-Producing Enterobacteriaceae

ESBL-producing Enterobacteriaceae in Nile perch and water were screened on MacConkey agar (Oxoid Ltd.) supplemented with 2 mg/L of cefotaxime (Moremi et al., 2016b). Briefly, 1 ml of an overnight enriched sample in BPW at 37°C was added to the MacConkey agar plates by the pour plate method and incubated overnight at 37°C (CLSI, 2016). Then, colonies with characteristics of Enterobacteriaceae were selected for ESBL confirmation using the double disk synergy method (Drieux et al., 2008; CLSI, 2016). The disks used were ceftazidime (30 μg) and cefotaxime (30 μg) (HiMedia Laboratories Pvt. Ltd., Mumbai, India), which were placed 20 mm apart from the center of a Mueller–Hinton agar (MHA) plate (Oxoid Ltd.) where amoxicillin/clavulanic acid (20 μg/10 μg) disk was placed. Plates were incubated at 37°C for 24 h. An inhibition zone around any of the disks under test increasing toward amoxicillin/clavulanic acid disk was interpreted as a presumptive ESBL-producing isolate, and the bacterial species were subsequently identified using API 20E (bioMérieux, France) based on the analytical profile index. The confirmed ESBL-producing *E. coli* isolates were further analyzed by PCR targeting β-lactamases genes – *bla*_CTX–M_, *bla*_TEM_, and *bla*_SHV_ – in single multiplex reactions using primer sequences described by Nasreldin and Khaldoon (2015) to confirm their ESBL-producing status. The confirmed ESBL-producing isolates were used for further analyses.

### Antimicrobial Disk Susceptibility Testing

The ESBL-producing *E. coli* isolates were subjected to antimicrobial susceptibility testing using standard disk diffusion method on MHA following the protocol and recommended antimicrobials by CLSI (2016). The following antimicrobials were selected: ampicillin/cloxacillin (AX; 10 μg), amoxicillin/clavulanic acid (AMC; 30 μg), gentamicin (GEN; 10 μg), sulfamethoxazole–trimethoprim (SXT; 25 μg), ciprofloxacin (CIP; 5 μg), cefotaxime (CTX; 30 μg), chloramphenicol (CHL; 30 μg), nalidixic acid (NAL; 30 μg), ceftazidime (CAZ; 30 μg), erythromycin (ERY; 15 μg), imipenem (IMP; 10 μg), and tetracycline (TET; 30 μg) (HiMedia Laboratories Pvt.). Although CLSI does not recommend the inclusion of erythromycin and ampicillin/cloxacillin, these antibiotics are commonly prescribed in Tanzania and were therefore included in the testing. *E*. *coli* ATCC 25922 was used as a quality control.

### DNA Extraction and Whole Genome Sequencing

DNA was extracted from the 11 ESBL-producing *E. coli* isolates using Maxwell RSC culture cell’s DNA kit following the manufacturer’s protocol in the automated Maxwell RSC instrument (Promega, Fitchburg, WI, United States). Genomes were sequenced at the 250-bp paired-end-read format using Nextera XT kit and the MiSeq instrument (Illumina, Inc., San Diego, CA, United States). The average coverage of WGS and read length are indicated in Supplementary Table 3. Raw sequence reads have been submitted to the European Nucleotide Archive (ENA) under the project number PRJEB34642 with the accession numbers of each sample available in Supplementary Tables S1, S3 .

### Data Analysis

Data collected were entered and stored into Microsoft Excel version 2010 (Microsoft Ltd., United States), and the prevalence of ESBL-producing *E. coli* in water and Nile perch at each sampling point was determined. *E. coli* genomes were *de novo* assembled using SPAdes 3.9.0 (Bankevich et al., 2012). Further analyses were performed using various web bioinformatics tools from the Center for Genomic Epidemiology (CGE),^2^ Enterobase,^3^ and BLASTn at National Centre for Biotechnology Information (NCBI).^4^ Plasmid replicons were detected using PlasmidFinder v2.0 (Carattoli et al., 2014), whereas virulence genes were determined using VirulenceFinder v.2.0 (Joensen et al., 2014), and *In Silico* analysis of resistance genes using ResFinder v2.2 (Zankari et al., 2012) from CGE using default settings.

Resistance to heavy metals and detergents was assessed using MyDbFinder 1.2 where our genomes were analyzed against plasmid-mediated genes such as the tellurite resistance gene *teh*A (NC_000913.3), the detergent-resistant phospholipase A, *pld*A (NC_003198.1), and the quaternary ammonium compound efflux *qacEdelta* (NG_048042.1) (Moremi et al., 2016b). Further heavy metal resistance operons encoding resistance to copper, cobalt, zinc, cadmium, magnesium, mercury, and chromium were analyzed through the subsystem annotation in RAST^5^ according to Brettin et al. (2015). The contigs harboring these genes and their positions on the contigs were compared with contigs harboring plasmids in the strains to confirm their location in the genomes. The location of β-lactamase genes on plasmids or chromosomes was determined by analyzing the contigs harboring the *bla*_*CTX–M–*__15_ and *bla*_*TEM–*__1__*B*_ using BLASTn in NCBI (Zhang et al., 2000).

Sequence types of the isolates were detected using *In Silico* MLST typing tool based on the seven housekeeping genes—*adk*, *fum*C, *gyr*B, *icd*, *mdh*, *pur*A, and *rec*A—for *E. coli* in Enterobase v.1.1.2 (Wirth et al., 2006). The phylogenetic analysis was performed using CSI Phylogeny v1.4 (Kaas et al., 2014) with the default options. The phylogenetic analysis included strains from this study and publicly available genomes of *E. coli* isolated from humans, foods, and the environments of the same sequence types as those identified for our strains. In addition to sequence type similarities, strains with the same serotype as our isolates were an added criterion in the selection of public genomes for phylogeny analysis. *E. coli* strain K12 sub-strain MG1655 (accession number SAMN02604091) was used as reference to root the tree. The list of *E. coli* strains used in phylogenetic analysis with their accession numbers is indicated in the table (Supplementary Table S1). The newick file of the tree was downloaded and edited using iTOL v4^6^ (Letunic and Bork, 2016). The pairwise SNP difference data can be found in a table (Supplementary Table S2).

## Results

### Prevalence of Extended-Spectrum β-Lactamase-Producing *Escherichia coli* in Nile Perch and Water

The overall prevalence of ESBL-producing *Escherichia coli* in Nile perch was 4.4% (8/180), whereas only one water sample collected offshore in the lake contained ESBL-producing *E. coli*. In this study, no other ESBL producers except for *E. coli* were detected. One Nile perch sample 1.7% (1/60) caught at fishing grounds offshore contained ESBL-producing *E. coli*, whereas two fish samples from landing sites contained ESBL-producing *E. coli* 3.3% (2/60) as did five fish samples purchased in domestic fish markets 8.3% (5/60) (Table 1).

**TABLE 1:** Prevalence of ESBL-producing *Escherichia coli* in water and Nile perch from Lake Victoria, Tanzania.

Origin	Sample type	Subsample type
Fishing grounds	Nile perch 1/60 (1.7%)	Fish gills 1/60 (1.7%)
	Water 1/60 (1.7%)	N/A
Landing sites	Nile perch 2/60 (3.3%)	Fish intestines 1/60 (1.7%)
		Fish surface mucus 2/60 (3.3%)
Markets	Nile perch 5/60 (8.3%)	Fish intestines 4/60 (6.7%)
		Fish gills 2/60 (3.3%)

*N/A, not applicable; ESBL, extended-spectrum β-lactamase.*

### Genomic Characteristics and Phylogenetic Analysis of Extended-Spectrum β-Lactamase-Producing *Escherichia coli* From Nile Perch

Analysis of the genome sequences from the 11 ESBL-producing *E. coli* revealed three clusters (C1, C2, and C3) separated by unique sequence type, serotype, phylogroup and also virulence and resistance genes patterns (Tables 2, 3). The cluster C1 included five clonal related strains (Z1 to Z5) with a SNP difference ranging from 3 to 13, all belonging to ST167, phylogroup A, and having serotype O9:H21. These strains possessed only one virulence gene (*gad)* and were isolated from fish and water samples collected at offshore fishing grounds, as well as from fish purchased at the landing sites. Subsample Z2 of mucus from the fish surface and Z3 (fish intestine) was from

**TABLE 2:** Genomic characterization of ESBL-producing *Escherichia coli* isolated in Nile perch.

Code	Origin	Sample type^a^	MLST	Serotype	Phylogroup	Virulence genes^b^
Z1	Landing site	Fish surface	167	O9:H21	A	*gad*
Z2	Landing site	Fish surface	167	O9:H21	A	*gad*
Z3	Landing site	Fish intestines	167	O9:H21	A	*gad*
Z4	Fishing grounds	Water	167	O9:H21	A	*gad*
Z5	Fishing grounds	Fish gills	167	O9:H21	A	*gad*
Z6	Markets	Fish intestines	636	O45:H7	B2	*gad*, *nfaE*, *iss*, *vat*
Z7	Markets	Fish intestines	636	O45:H7	B2	*gad*, *nfaE*, *iss*, *vat*
Z8	Markets	Fish gills	156	ONT:H28	B1	*gad*, *iss*, *lpfA*
Z9	Markets	Fish gills	156	ONT:H28	B1	*gad*, *iss*, *lpfA*
Z10	Markets	Fish intestines	636	O45:H7	B2	*gad*, *nfaE*, *iss*, *vat*
Z11	Markets	Fish intestines	636	O45:H7	B2	*gad*, *nfaE*, *iss*, *vat*

*ESBL, extended-spectrum β-lactamase. ^*a*^Subsamples Z2 and Z3 from a landing site harbored two ESBL producers in mucus from the fish surface and fish intestine. Likewise, subsamples Z9 and Z10 from a market harbored two different ESBL producers isolated from fish intestines and gills. ^*b*^*gad*, glutamate decarboxylase; *nfaE*, diffuse adherence fimbrillar adhesin gene; *iss*, increased serum survival; *vat*, vacuolating autotransporter toxin; and *lpfA*, long polar fimbriae.*

**TABLE 3:** Phenotypic and genotypic antimicrobial resistance of ESBL-producing *Escherichia coli* isolates.

Code	Aminoglycoside	Sulfonamide–trimethoprim	Fluoroquinolone	Tetracycline	Macrolide	β-Lactamases	Chloramphenicol
Z1	GEN/+; *aad*A2, *aac(3)*-*IId*	SXT/+; *sul*1, *sul*2, *dfr*A12	CIP, NAL/+; *qep*A4	TET/+; *tet*B, *tet*D	−/; *mph*A, *mdf*A	AX, CTX/+; CTX-M-15, TEM-1B	CHL/+; *cat*A1
Z2	−/; *aad*A2, *aac(3)-IId*	SXT/+; *sul*1, *sul*2, *dfr*A12	CIP, NAL/+; *qep*A4	TET/+; *tet*B, *tet*D	*−/*; *mph*A, *mdf*A	AX, CTX/+; CTX-M-15, TEM-1B	CHL/+; *cat*A1
Z3	−/; *aad*A2, *aac(3)*-*IId*	SXT/+; *sul*1, *sul*2, *dfr*A12	CIP, NAL/+; *qep*A4	TET/+; *tet*B, *tet*D	ERY/+; *mph*A, *mdf*A	AX/+; CTX-M-15, TEM-1B	CHL/+; *cat*A1
Z4	−/; *aad*A2, *aac(3)-IId*	SXT/+; *sul*1, *sul*2, *dfr*A12	CIP, NAL/+; *qep*A4	TET/+; *tet*B, *tet*D	*-/*; *mph*A; *mdf*A	AX, CTX/+; CTX-M-15, TEM-1B	CHL/+; *cat*A1
Z5	−/; *aad*A2, *aac(3)-IId*	SXT/+; *sul*1, *sul*2, *dfr*A12	CIP, NAL/+; *qep*A4	TET/+; *tet*B, *tet*D	ERY/+; *mph*A, *mdf*A	AX, CTX/+; CTX-M-15, TEM-1B	CHL/+; *cat*A1
Z6	GEN/+; *aad*A1, *str*A, *str*B	SXT/+; *sul*2, *dfr*A1	ND	−/; *tet*B	ERY/+; *mph*A, *mdf*A	AX, CTX/+; CTX-M-15, TEM-1B	ND
Z7	GEN/+; *aad*A1, *str*A, *str*B	SXT/+; *sul*2, *dfr*A1	ND	TET/+; *tet*B	ERY/+; *mph*A, *mdf*A	AX, CTX/+; CTX-M-15, TEM-1B	ND
Z8	ND	SXT/+; *sul*1, *dfr*B4	CIP, NAL/+; *qep*A4	TET/+; *tet*B	ERY/+; *mph*A, *mdf*A	AX, CTX/+; CTX-M-15, TEM-1B	CHL/+; *cat*A1
Z9	ND	SXT/+; *sul*1, *dfr*B4	CIP, NAL/+; *qep*A4	TET/+; *tet*B	ERY/+; *mph*A, *mdf*A	AX/+; CTX-M-15, TEM-1B	CHL/+; *cat*A1
Z10	GEN/+; *aad*A1, *str*A, *str*B	SXT/+; *sul*2, *dfr*A1	ND	TET/+; *tet*B	ERY/+; *mph*A, *mdf*A	AX, CTX/+; CTX-M-15, TEM-1B	ND
Z11	GEN/+; *aad*A1, *str*A, *str*B	SXT/+; *sul*2, *dfr*A1	ND	TET/+; *tet*B	ERY/+; *mph*A, *mdf*A	AX, CTX/+; CTX-M-15, TEM-1B	ND

*AX, ampicillin/cloxacillin; AMC, amoxicillin/clavulanic acid; CAZ, ceftazidime; CIP, ciprofloxacin; CHL, chloramphenicol; CTX, cefotaxime; ERY, erythromycin; GEN, gentamicin; IMP, imipenem; NAL, nalidixic acid; SXT, sulfamethoxazole–trimethoprim; and TET, tetracycline; ESBL, extended-spectrum β-lactamase. ND, no phenotypic and genotypic resistance; +, phenotypic resistance; −, no phenotypic resistance.*

the same fish sampled at a landing site, which harbored two ESBL producers. Likewise, subsamples Z9 and Z10 from the same fish collected at a market harbored two different ESBL producers isolated from fish intestines and gills. Two clonal strains (Z8 and Z9) formed cluster C2 with 11 SNPs apart. They belong to ST156, phylogroup B1, and showed serotype ONT:H28 and were both isolated from Nile perch obtained at local fish markets. These two strains contained additional virulence genes including *gad*, *iss*, *lpf*A, and *nfa*E.

Strains Z6, Z7, Z10, and Z11 isolated from Nile perch from fish markets formed the phylogenetic cluster C3 and had a SNP difference ranging from 4 to 17. These four strains belong to ST636, phylogroup B2; have serotype O45:H7; and harbor the *vat* gene in addition to all the virulence genes present in C2 strains. Among these isolates, we noted that one fish sample harbored two different ESBL producers (ST156 and ST636) in different sample subtypes, that is, from fish intestines and gills.

**FIGURE 1:**
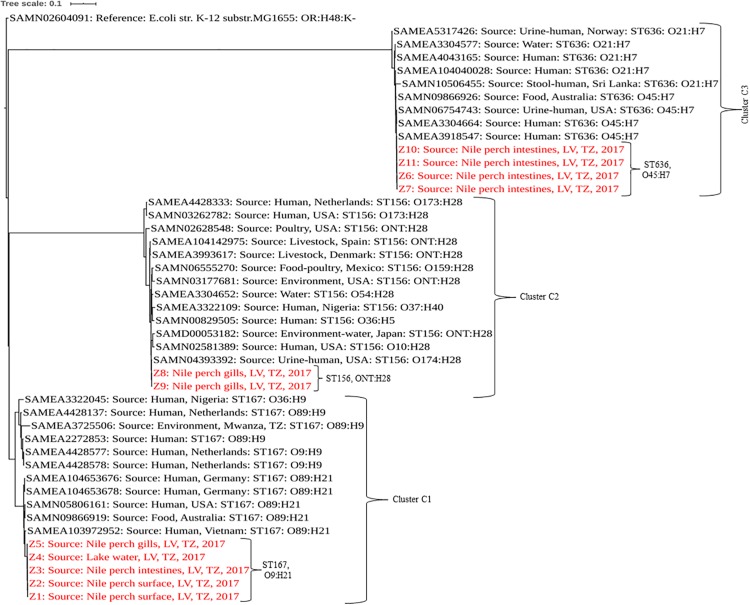
Phylogenetic tree based on SNPs showing clonal relationship among ESBL-producing E. coli isolated from Nile perch and global public genomes of E. coli strains. Our strains are highlighted in red color. LV, Lake Victoria; TZ, Tanzania.

In a comparison with *E. coli* isolated from humans, animals, and the environment globally, our strains showed a sequence type-based clustering where the publically available ST167 genomes clustered with our C1 strains, as did the publically available ST156 and ST636 genomes with our C2 and C3 strains, respectively. However, we recorded some wide within-clade variations between our strains and the public genomes of the same STs with up to 5,104, 2,440, and 1,391 SNP differences in C1, C2, and C3, respectively, (Figure 1 and Supplementary Table S2).

### Antimicrobial Resistance, Plasmid Replicon Profiles, Heavy Metal, and Detergent Resistance

Overall, the genotype was consistent with the phenotypic resistance patterns, as well as occurrence of resistance genes and plasmid replicon types in the 11 ESBL-producing *E. coli*, which showed a similar clustering pattern as shown above (Tables 3, 4; Supplementary Table S3). The isolates showed phenotypic antimicrobial resistance to sulfamethoxazole–trimethoprim (100%), ampicillin/cloxacillin (100%), erythromycin (72.7%; 8/11), tetracycline (90.9%; 10/11), nalidixic acid (63.6%; 7/11), and chloramphenicol (63.6%; 7/11) (Table 3). C1 strains showed phenotypic and genotypic resistances to aminoglycosides [*aad*A2 and *aac(3)*-*IId*], sulfonamide–trimethoprim (*sul*1, *sul*2, and *dfr*A12), fluoroquinolone (*qep*A4), tetracycline (*tet*B and *tet*D), macrolides (*mph*A and *mdf*A), chloramphenicol (*cat*A1), and the β-lactams ampicillin/cloxacillin and cefotaxime (*bla*_CTX–M–__15_ and *bla*_TEM–__1__B_). However, strains Z1, Z2, and Z4 of C1 did not show phenotypic resistance to erythromycin despite the presence of *mph*A and *mdf*A conferring resistance to macrolides. All C1 strains have the plasmid replicon types IncFIA, IncFIB, IncFII, IncX1, Col8282, and Col156. Isolates contained IncF plasmids with the pMLST type [F48:A1:B49]. Strains Z8 and Z9 of clade C2 showed resistance to sulfonamide–trimethoprim (*sul*1 and *dfr*B4), fluoroquinolone (*qep*A4), tetracycline (*tet*B), macrolides (*mph*A and *mdf*A), chloramphenicol (*cat*A1), and the β-lactams ampicillin/cloxacillin and cefotaxime (*bla*_CTX–M–__15_ and *bla*_TEM–__1__B_). They were susceptible to aminoglycosides and only possessed one plasmid replicon type Col440I harboring the β-lactam genes.

**TABLE 4:** Plasmid profiles and location of the β-lactam genes in ESBL-producing *Escherichia coli.*

Node	Plasmid replicon types	pMLST	Location of ESBL and other β-lactamase genes
Z1	IncFIA; IncFIB; IncFII; IncX1; Col8282; Col156	IncF [F48:A1:B49]	Plasmid (CTX-M-15: TEM-1B)
Z2	IncFIA; IncFIB; IncFII; IncX1; Col8282; Col156	IncF [F48:A1:B49]	Plasmid (CTX-M-15: TEM-1B)
Z3	IncFIA; IncFIB; IncFII; IncX1; Col8282; Col156	IncF [F48:A1:B49]	Plasmid (CTX-M-15: TEM-1B)
Z4	IncFIA; IncFIB; IncFII; IncX1; Col8282; Col156	IncF [F48:A1:B49]	Plasmid (CTX-M-15: TEM-1B)
Z5	IncFIA; IncFIB; IncFII; IncX1; Col8282; Col156	IncF [F48:A1:B49]	Plasmid (CTX-M-15: TEM-1B)
Z6	IncFIA; IncFIB; IncFII; IncQ1; ColRNAI; Col (BS512)	IncF [F1:A1:B1]	Chromosome (CTX-M-15)
			Plasmid (TEM-1B)
Z7	IncFIA; IncFIB; IncFII; IncQ1; ColRNAI; Col (BS512)	IncF [F1:A1:B1]	Chromosome (CTX-M-15)
			Plasmid (TEM-1B
Z8	Col440I	N/A	Plasmid (CTX-M-15: TEM-1B)
Z9	Col440I	N/A	Plasmid (CTX-M-15: TEM-1B)
Z10	IncFIA; IncFIB; IncFII; IncQ1; ColRNAI; Col (BS512)	IncF [F1:A1:B1]	Chromosome (CTX-M-15)
			Plasmid (TEM-1B)
Z11	IncFIA; IncFIB; IncFII; IncQ1; ColRNAI; Col (BS512)	IncF [F1:A1:B1]	Chromosome (CTX-M-15)
			Plasmid (TEM-1B)

*ESBL, extended-spectrum β-lactamase.*

The C3 strains were susceptible to fluoroquinolones and chloramphenicol but showed resistance to aminoglycosides (*aad*A1, *str*A, and *str*B), sulfonamide–trimethoprim (*sul*2 and *dfr*A1), tetracycline (*tet*B), macrolides (*mph*A and *mdf*A), and the β-lactams ampicillin/cloxacillin and cefotaxime (*bla*_CTX–M–__15_ and *bla*_TEM–__1__B_). Despite the presence of *tet*B, strain Z6 did not show phenotypic resistance to tetracycline. All C3 strains have plasmid replicon types IncFIA, IncFIB, IncFII, IncQ1, ColRNAI, and Col (BS512) with the IncF harboring the *bla*_TEM–__1__B_ gene, and they carried the *bla*_CTX–M–__15_ on their chromosome. Isolates in this cluster contained IncF plasmids with the pMLST-type IncF [F1:A1:B1]. Strains in all three clusters were sensitive to imipenem.

All cluster C1 and C2 strains harbored the plasmid-mediated detergent resistance gene, *qacEdelta*, encoding resistance to quaternary ammonium compounds. All the strains contained resistance genes to copper, cobalt, zinc, cadmium, magnesium, mercury, and tellurite, which were chromosomally located (Supplementary Table S4). However, chromium resistance was only recorded in strains of cluster C1 isolated from fishing grounds and landing sites.

## Discussion

The occurrence of ESBL-producing *Escherichia coli* was low in Nile perch and water samples collected at offshore fishing grounds and at landing sites of Lake Victoria but higher in fish from local fish markets in Mwanza. ESBL-producing *E. coli* have been reported in different wild-caught fish species in Switzerland, Algeria, and India (Abgottspon et al., 2014; Singh et al., 2017; Dib et al., 2018), as well as in farmed fish in China (Jiang et al., 2012) and Egypt (Ishida et al., 2010). Moremi et al. (2016b) reported a slightly higher prevalence of ESBL-producing bacteria in wild-caught tilapia sold at markets in Mwanza (13.3%) as compared with our findings in Nile perch (8.3%). Diverse bacterial species were found in the tilapia (Moremi et al., 2016b), whereas only *E. coli* was isolated in our Nile perch. This is probably because tilapia is usually found in shallow water with relatively high levels of fecal pollution and hence higher possibility of finding a variety of Enterobacteriaceae than finding these bacteria in the Nile perch, which are caught in deep water with lower pollution levels. Thus, these studies suggest that the aquatic environment and fish are likely sources and dissemination routes for ESBL-producing bacterial species and their resistance genes, although their relative importance from a public health perspective is uncertain.

The low prevalence of ESBL-producing *E. coli* in Nile perch from fishing grounds is likely due to the fact that these fish are commonly found and caught in deep water far away from the lake shore where fecal pollution is low compared with the shallow water near the shore of the lake (Baniga et al., 2019). Any occurrence of ESBL-producing *E. coli* in Nile perch caught offshore could be attributed to their eating habits feeding on other fish species, for example, tilapia and sardines, which are found in shallow water with higher fecal contamination levels. This hypothesis is corroborated by the findings of Moremi et al. (2016b) where gut intestinal samples from tilapia contained ESBL-producing bacteria. Because boats of artisanal small-scale fishers do not have toilet facilities, the fishermen have to defecate into the lake, with a potential introduction of ESBL-producing *E. coli* into the lake environment.

ESBL-producing *E. coli* isolated in Nile perch from markets belonged to clusters C2 and C3 and seem to harbor more virulence genes than do isolates originating from fish caught offshore. This higher pathogenicity indicates a human origin, and their occurrence in fish sold at markets is likely caused by fecal cross-contamination from fish handlers and vendors. This is further supported by the fact that most of the market strains of C3 belong to phylogroup B2, which is usually composed of extra-intestinal pathogenic *E. coli* strains (Abgottspon et al., 2014; Huang et al., 2016; Sánchez-Benito et al., 2017). It is important to note that our prevalence of ESBL-producing *E. coli* are generally very low, not allowing for any statistical comparison between fish of different origins. Moreover, possible associations between occurrence of ESBL-producing *E. coli* in fish and human handling activities can be better studied through comparative genomic investigations including clinical human isolates and our environmental strains.

The virulence gene *gad* harbored by C1 strains is an important gene for both commensal and pathogenic enteric bacteria especially *E. coli*, as the reaction products of *gad* are essential for survival in an acidic environment and for successful colonization of the host cell (Tramonti et al., 2002). The additional virulence genes shown by C2 and C3 strains included *nfa*E, which is essential for diffuse adherence fimbrillar adhesin; *iss* plays a significant role in increased serum survival; and the *vat* gene is important for vacuolating autotransporter toxin in host cells for pathogenicity processes. These virulence genes were previously reported from *E. coli* in humans as well as from pigs and bovine (Szmolka et al., 2012; Madoshi et al., 2016; Ahmed et al., 2017). The *lpf*A gene is important for long polar fimbriae and has been commonly associated with the ability to invade epithelial cells in animals and humans (Dogan et al., 2012).

Our strains were epidemiologically unrelated, but their grouping into three clusters with further clustering with other *E. coli* strains of the same STs isolated from human, food, and environmental samples around the globe underlines the transmission potential of the ESBL genes across various niches and locations favored by plasmids that they harbor. Our strains of cluster C1 (ST167) belong to the phylogroup A known to contain commensal strains (Abgottspon et al., 2014; Huang et al., 2016; Sánchez-Benito et al., 2017), and this is supported by the presence of only one virulence gene (*gad*). Moremi et al. (2016b) reported a single environmental sample from Mwanza containing an ESBL-producing *E. coli* of ST167, belonging to the newly defined phylogroup E harboring *bla*_CTX–M–__15_ and *bla*_*OXA–*__1_. This ST167 strain of phylogroup E differed from our strains of ST167 of phylogroup A with up to 5,104 SNPs. The variation among strains of the same STs can be attributed to the differences in serotype as well as their genetic content such as the differences in the ESBL genes that they contained. Our study corroborates earlier findings that *bla*_CTX–M–__15_ is the predominant ESBL gene in Enterobacteriaceae in Tanzania (Moremi et al., 2016b), yet the sources are still unknown. ESBL-producing *E. coli* ST167 have been previously reported isolated in humans in China and Spain carrying *bla*_CTX–M–__15_ and *bla*_TEM–__1_ genes (Huang et al., 2016; Sánchez-Benito et al., 2017). This substantiates the possibilities of horizontal gene transfer of the ESBL genes from food products or the environment to humans where bacterial pathogens can acquire them. Such transmission is favored by plasmids on which the resistance genes are located (Anjum et al., 2019) as is the case for the *bla*-_*CTXM–*__15_ and *bla*_TEM–__1_ genes, which were located on the IncF plasmid (Table 4). We observed the plasmid IncF [F1:A1:B1] within all four C3 strains, which had the same ST636 and the same pMLST and showed a SNP difference ranging from 4 to 17. The C3 strains all came from intestinal samples, and we cannot explain their clonal nature as the origin and previous exposures of the fish purchased at the markets are unknown. The IncF [F48:A1:B49] was shown in all five C1 strains originating from intestinal and surface mucus samples of fish from one landing site as well as from fish (gills) and water collected offshore. Similarly to C3 strains, the C1 strains were epidemiologically unrelated, and we are not able to explain the clonal nature of the C1 strains. In contrast to our study, the ESBL-producing bacteria reported in tilapia from the Mwanza region by Moremi et al. (2016b) included a much more diverse population of bacterial species, a variety of ESBL genes, and no indication of a clonal relationship. The occurrence of genes encoding for resistance to metals such as copper, cobalt, zinc, cadmium, and mercury, and also genes encoding resistance to detergents is similar to previous findings in fish and aquatic environment of the Lake Victoria; the genes have been reported to play a role in environmental persistence support in bacterial survival (Moremi et al., 2016b; Hounmanou et al., 2019). Most of metal resistance genes were located on the chromosome, whereas ESBL genes were mostly located on the plasmids, except that all four ST636 isolates harbored a chromosomally located *bla*_CTX–M–__15_ gene, and it is uncertain to what extent exposure to metals, for example, used as livestock feed additives and as pollutants in aquatic environments, may play a role as co-selectors of ESBL resistance.

In addition to the ESBL genes, our strains are all multidrug resistant despite their relatively low virulence (Magiorakos et al., 2012). The resistance genes found have been frequently reported in Gram-negative bacteria, and because the genes are often plasmid mediated, they may be circulated horizontally among different bacterial species (van Hoek et al., 2011). These additional resistances shown by the ESBL-producing *E. coli* may also reflect the frequent use of antimicrobials reported in human and veterinary medicine in the Mwanza region (Moremi et al., 2016a; Seni et al., 2016). The location of the additional resistance genes requires further analysis and depiction of the genetic environment of *bla*_CTX–M__15_ genes in the *bla*_CTX–M–__15_-encoding isolates (Moremi et al., 2016b).

Some strains in C1 and C3 did not show phenotypic resistance to antimicrobials that they harbored resistance genes for, that is, erythromycin and tetracycline. This is an increasing observation in genomic studies and could be due to various factors including the concentration and quality of the antimicrobial disks (Eze et al., 2014) or intrinsic factors inhibiting the expression of the resistance genes (Weill et al., 2017). This has also been associated with random mutations, which could be accumulated in gene sequences encoding resistance to some antimicrobials (Davis et al., 2011; Hussain et al., 2014).

## Conclusion

In conclusion, we report a very low prevalence of ESBL-producing *E. coli* in Nile perch from Lake Victoria. Our data suggest that as far as ESBL-producing enteric bacteria are concerned, the consumption of Nile perch represent limited food safety risks compared with other human exposures to ESBL-producing *E. coli*, for example, through direct human-to-human fecal transmission and consumption of livestock meat products. The grouping of the 11 ESBL-producing *E. coli* into three clades each showing identical characteristics, for example, STs, phylogroup, antimicrobial resistance, and virulence genes, is surprising, and we are not able to explain the clonal nature of these clades as the *E. coli* strains were epidemiologically unrelated. All isolates harbored *bla*_CTX–M__–__15_ and *bla*_TEM__–__1_ genes together with additional antimicrobial and detergent resistance genes carried by the common plasmids replicon types IncF, IncX, IncQ, and Col. Further studies are needed to determine the role of fish and aquatic environments as sources of ESBL-producing bacteria and resistance genes including the importance of fecal pollution sources, for example, discharge of sewage and runoff water from fertilized agricultural soil, as well as the ecology of such resistant bacteria in aquatic environments.

Our study has some limitations, including failure to obtain other Enterobacteriaceae spp. than *E. coli.* This is likely because of the selective isolation procedure used with supplement of cefotaxime to obtain ESBL-producing strains; thus, most of other bacteria present both in deep water and in fish from markets were likely sensitive to cefotaxime and did therefore not grow on the MacConkey agar. Although *E. coli* isolated from Nile perch at fish markets were of different sequence types and harbored more virulence genes than did isolates from deep water, a comparison study with ESBL-producing *E. coli* from clinical specimens with environmental strains may provide further information about transmission with the aquatic environment, fish, and human compartments.

## Data Availability Statement

Raw sequence reads have been submitted to the European Nucleotide Archive (ENA) under the project number PRJEB34642.

## Ethics Statement

The present study required no ethical approval because we did not manipulate any live fish in the study. Fish samples used in the study were fished by fishermen and constitute dead but fresh fish products ready for marketing and consumption. We purchased these fish from the fishermen, in boats at landing sites and in the markets from fish retailers as indicated in the manuscript like any other fish consumer, and then we brought them to the laboratory for analyses.

## Author Contributions

ZB collected samples, carried out laboratory and data analysis, and wrote the draft manuscript. YH participated in laboratory analysis and performed genomic data analysis and editing and critical review of the manuscript. EK participated in genomic data analysis and reviewed the manuscript. LK and RM supervised the study and were involved in reviewing the manuscript and facilitation of resources for the study. AD supervised the study and provided guidance and resources, contributed in genomic data analysis, revised the manuscript, and gave the final approval of the manuscript. All authors read and approved the final manuscript for submission.

## Conflict of Interest

The authors declare that the research was conducted in the absence of any commercial or financial relationships that could be construed as a potential conflict of interest.

## Footnotes

1 www.seward.co.uk

2 https://cge.cbs.dtu.dk/services/

3 http://enterobase.warwick.ac.uk/

4 https://www.ncbi.nlm.nih.gov/

5 http://rast.nmpdr.org/rast.cgi

6 https://itol.embl.de/
